# Research Progress on the Mechanisms of Gut Microbiota Dysbiosis Associated With Idiopathic Pulmonary Fibrosis: A Review

**DOI:** 10.7759/cureus.102429

**Published:** 2026-01-27

**Authors:** Xiaolong Li, Shuhao Xu, Yi Li, Rongli Wang, Chao Qin, Xin Wang

**Affiliations:** 1 Pulmonary and Critical Care Medicine, Deyang People's Hospital, Deyang, CHN; 2 Stomatology, Deyang People's Hospital, Deyang, CHN; 3 Pulmonary and Critical Care Medicine, The Affiliated Hospital of Southwest Medical University, Luzhou, CHN

**Keywords:** gut-lung axis, gut microbiota, idiopathic pulmonary fibrosis, immune modulation, microbial metabolites, therapeutic targets

## Abstract

Idiopathic pulmonary fibrosis (IPF) is a chronic, progressive fibrotic interstitial lung disease with an incompletely understood pathogenesis. In recent years, growing evidence has highlighted the critical role of gut microbiota dysbiosis in the onset and progression of IPF. This review comprehensively summarizes the characteristics of gut microbiota alterations associated with IPF, explores the underlying mechanisms driving these changes, and examines their impact on disease development. Particular emphasis is placed on the emerging concept of the “gut-lung axis,” which elucidates the bidirectional communication between the intestinal microbiome and pulmonary health. The review further discusses microbial metabolites and immune modulation as key mediators linking gut dysbiosis to pulmonary fibrosis. Additionally, current advances in microbiota-targeted therapeutic strategies, including probiotics, prebiotics, and fecal microbiota transplantation, are analyzed for their potential in IPF management. By systematically integrating recent findings, this article aims to deepen the understanding of IPF pathophysiology and provide a theoretical foundation for novel treatment targets centered on gut microbiota regulation.

## Introduction and background

Idiopathic pulmonary fibrosis (IPF) is a chronic, progressive interstitial lung disease characterized by irreversible lung function decline and poor prognosis, with a median survival of only three to five years following diagnosis. The etiology of IPF remains largely unknown, complicating treatment options and highlighting the need for a deeper understanding of its underlying mechanisms. Traditionally, IPF has been viewed primarily as a pulmonary disease; however, emerging research suggests that it may also be influenced by systemic factors such as gut microbiota dysbiosis. The gut microbiota, a complex community of microorganisms residing in the gastrointestinal tract, plays a crucial role in modulating immune responses, inflammation, and metabolism, which are all critical in the pathogenesis of various diseases, including respiratory conditions like IPF [1].

The concept of the “gut-lung axis” has gained traction in recent years, emphasizing the bidirectional communication between the gut and the lungs. This interaction suggests that alterations in gut microbiota can influence lung health and disease progression. For instance, dysbiosis, or an imbalance in the gut microbiota, has been linked to aberrant immune responses and increased inflammation, both of which are pivotal in the development and exacerbation of IPF [1]. Recent studies have shown that patients with chronic respiratory diseases, including IPF, exhibit significant changes in both gut and lung microbiota composition, which may contribute to disease severity and progression [2]. This highlights the importance of understanding the mechanisms by which gut microbiota may influence lung pathology, particularly in the context of IPF.

Research has demonstrated that gut microbiota can affect lung function through several mechanisms, including the production of metabolites, modulation of immune responses, and regulation of systemic inflammation. For example, metabolites produced by gut bacteria, such as short-chain fatty acids (SCFAs), have been shown to exert anti-inflammatory effects and support immune homeostasis [3]. Additionally, the gut microbiota can influence the integrity of the gut-lung barrier, which is crucial for maintaining lung health and preventing inflammatory responses that can lead to fibrosis [1]. Understanding these pathways is essential for elucidating the role of gut microbiota in the pathogenesis of IPF and may provide new therapeutic avenues for managing this debilitating disease.

Despite the growing body of evidence linking gut microbiota dysbiosis to respiratory diseases, the specific mechanisms by which these alterations contribute to IPF remain incompletely understood. Current research is focused on identifying key microbial species and their metabolites that may play a role in modulating lung inflammation and fibrosis [4]. Furthermore, therapeutic strategies aimed at restoring gut microbiota balance, such as the use of probiotics, prebiotics, and fecal microbiota transplantation (FMT), are being explored for their potential to mitigate the effects of IPF [1]. This review aims to systematically summarize the characteristics of gut microbiota dysbiosis associated with IPF, elucidate the mechanisms involved, and discuss the clinical implications of these findings, ultimately highlighting the potential for microbiota-targeted therapies in the management of IPF.

## Review

IPF patients’ gut microbiota characteristics

Changes in Gut Microbiota Composition

IPF patients exhibit significant alterations in their gut microbiota composition, characterized by reduced microbial diversity and specific shifts in bacterial taxa abundance. Studies have consistently reported a marked decrease in alpha diversity, reflecting a loss of richness and evenness within the gut microbial community in IPF compared to healthy controls. This diminished diversity is accompanied by notable compositional changes, including a decreased proportion of Bacteroidetes and an increased abundance of Firmicutes, resulting in an abnormal Firmicutes/Bacteroidetes (F/B) ratio. Such dysbiosis is critical because these phyla are key players in maintaining gut homeostasis and immune modulation. At the genus level, SCFA-producing bacteria such as Prevotella and Roseburia are significantly reduced in IPF patients. These genera are important for producing butyrate and other SCFAs, which have anti-inflammatory properties and support epithelial barrier integrity. Conversely, potentially pathogenic bacteria like Escherichia coli and Clostridium difficile show excessive proliferation in the gut of IPF patients, potentially contributing to systemic inflammation and immune dysregulation. Animal models of pulmonary fibrosis induced by bleomycin have demonstrated parallel changes, with gut microbiota shifts correlating with lung pathology and systemic markers of disease. These findings suggest that gut microbiota dysbiosis in IPF involves both a loss of beneficial commensals and an overgrowth of opportunistic pathogens, which may influence disease pathogenesis through the gut-lung axis. Moreover, clinical studies have identified that antifibrotic treatments can modulate gut microbiota composition, indicating a dynamic interplay between therapy and microbial ecology. Collectively, these compositional alterations in the gut microbiota of IPF patients underscore the potential role of microbial communities in disease development and progression, providing a foundation for exploring microbiota-targeted therapeutic strategies [1,5,6].

Correlation Between Gut Microbiota and Disease Severity

The degree of gut microbiota dysbiosis in IPF patients is closely correlated with clinical indicators of disease severity, including pulmonary function parameters such as forced vital capacity (FVC) and diffusing capacity for carbon monoxide (DLCO). Studies have demonstrated a negative correlation between microbial diversity and lung function; patients exhibiting more profound microbial imbalances tend to have worse pulmonary function and more advanced fibrosis. Specific microbial signatures have been proposed as predictive markers for disease progression and acute exacerbation risk. For example, reduced abundance of SCFA-producing bacteria like Faecalibacterium prausnitzii and Roseburia correlates with increased systemic inflammation and poorer outcomes. Conversely, enrichment of pro-inflammatory taxa, including Enterobacteriaceae, is associated with accelerated disease progression. The gut microbiota profile also shows potential as a prognostic biomarker; certain bacterial taxa and their metabolites, such as trimethylamine-N-oxide (TMAO), have been linked to fibrosis severity and mortality risk. Furthermore, metabolomic analyses reveal that gut microbiota alterations influence host metabolic pathways related to amino acid and lipid metabolism, which may modulate fibrotic processes. Experimental evidence from animal models supports these clinical observations, where gut microbial changes mediate immune dysregulation and fibrogenesis through the gut-lung axis. These microbial patterns offer a promising avenue for non-invasive disease monitoring and personalized therapeutic interventions. The integration of gut microbiota profiling into clinical practice could enhance prognostic accuracy and guide treatment decisions in IPF management [1,5].

Factors influencing gut microbiota dysbiosis

Influence of Drug Factors

Drug administration is a pivotal external factor that can significantly alter gut microbiota composition, thereby influencing the pathogenesis and progression of diseases such as IPF. Antifibrotic drugs commonly used in IPF treatment, including nintedanib and pirfenidone, have been implicated in modulating gut microbiota, although direct evidence remains limited. Recent clinical studies have shown that patients with IPF exhibit distinct gut microbiota profiles compared to healthy controls, characterized by altered abundance of specific bacterial taxa such as increased Enterobacterales and decreased beneficial genera like Bifidobacteriales and Ruminococcus [6]. While the effects of antifibrotic drugs on these microbial populations are not fully elucidated, a notable observation is the decreased abundance of Lachnospiraceae UCG 004 in patients receiving antifibrotic therapy, suggesting that these drugs may influence gut microbial ecology. Proton pump inhibitors (PPIs), frequently prescribed to IPF patients due to the high prevalence of gastroesophageal reflux disease (GERD), have also been associated with gut microbiota disturbances. PPIs can reduce gastric acidity, facilitating the survival and colonization of oral and upper gastrointestinal microbes in the gut, thereby contributing to dysbiosis. This is supported by studies demonstrating that PPI use correlates with reduced microbial diversity and overgrowth of potentially pathogenic bacteria in the gut, which may exacerbate IPF progression through systemic inflammation and immune modulation [3]. Antibiotic therapy, often employed to manage respiratory infections in IPF patients, represents another critical factor influencing gut microbiota. Antibiotics induce profound and sometimes prolonged dysbiosis by reducing microbial diversity and altering the balance between beneficial and harmful bacteria. Such perturbations can impair gut barrier integrity, promote systemic endotoxemia, and modulate immune responses, potentially worsening IPF outcomes. Experimental models have shown that antibiotic-induced dysbiosis leads to increased pro-inflammatory cytokines and compromised intestinal barriers, effects that may be reversed by interventions such as FMT or probiotics [7,8]. Moreover, antibiotics may reduce the abundance of bacteria involved in the production of SCFAs, metabolites known for their anti-inflammatory properties and role in maintaining epithelial homeostasis. Therefore, while antifibrotic drugs, PPIs, and antibiotics are indispensable in IPF management, their impacts on gut microbiota composition and function warrant careful consideration. Understanding these drug-microbiota interactions is essential for optimizing therapeutic strategies and mitigating adverse effects related to gut dysbiosis in IPF patients.

Role of Host Factors

Host intrinsic factors, including age, genetic predisposition, and comorbidities, play a crucial role in shaping the gut microbiota and influencing its dysbiosis in IPF. Aging is associated with significant alterations in gut microbial diversity and composition, often characterized by reduced beneficial bacteria and increased pro-inflammatory taxa. This age-related dysbiosis may contribute to the progression of IPF by promoting chronic systemic inflammation, impairing immune regulation, and disrupting the gut-lung axis. Studies in elderly populations have demonstrated that malnutrition and immunosenescence exacerbate these microbial shifts, further compromising respiratory health and increasing susceptibility to pulmonary fibrosis [9]. Genetic factors also modulate the interplay between host and microbiota. Notably, polymorphisms in the MUC5B gene, a major genetic risk factor for IPF, may influence mucosal barrier integrity and microbial colonization patterns in the gut and lungs. This genetic predisposition could alter host-microbiota interactions, leading to dysregulated immune responses and fibrosis development. Although direct evidence linking MUC5B polymorphisms to gut microbiota changes in IPF is limited, emerging research suggests that such genetic variants affect mucin production and composition, thereby shaping microbial communities and their metabolites [10]. Comorbid conditions prevalent in IPF patients, particularly GERD, have a significant impact on gut microbiota homeostasis. GERD can alter the gastrointestinal microenvironment through acid reflux and micro-aspiration, leading to changes in microbial composition and increased intestinal permeability. These alterations may facilitate translocation of microbial products and inflammatory mediators, exacerbating pulmonary inflammation and fibrosis. The use of PPIs to manage GERD further compounds these effects by modifying gastric pH and microbial survival [3]. Additionally, systemic inflammation and immune dysregulation associated with comorbidities such as cardiovascular disease and metabolic disorders contribute to gut microbial imbalance and barrier dysfunction. Collectively, host factors including aging, genetic susceptibility, and comorbidities create a milieu conducive to gut microbiota dysbiosis, which in turn influences IPF pathogenesis through complex gut-lung axis mechanisms. Elucidating these interactions offers potential avenues for personalized interventions targeting host and microbial factors to mitigate disease progression.

Potential mechanisms by which gut microbiota dysbiosis promotes IPF

Role of Microbial Metabolites

SCFAs, including acetate, propionate, and butyrate, are principal metabolites produced by gut microbial fermentation of dietary fiber and have been shown to exert significant immunoregulatory and anti-inflammatory effects. In the context of IPF, a reduction in SCFAs due to gut microbiota dysbiosis can impair these protective functions. SCFAs serve as energy substrates for intestinal epithelial cells and modulate immune responses by activating G protein-coupled receptors (GPCRs) such as FFAR2 and FFAR3, inhibiting histone deacetylases (HDACs), and influencing gene expression related to inflammation and fibrosis. The diminished SCFA levels observed in IPF patients correlate with increased systemic inflammation and may exacerbate fibrotic processes by promoting pro-inflammatory cytokine production and impairing regulatory T cell (Treg) differentiation. Additionally, SCFAs have been found to regulate key signaling pathways such as TGF-β1/Smad2/3, which are central to epithelial-to-mesenchymal transition (EMT) and fibrosis progression. Beyond SCFAs, abnormalities in tryptophan metabolism, another microbial metabolic pathway, affect the aryl hydrocarbon receptor (AhR) signaling. Dysregulated tryptophan catabolism leads to altered levels of metabolites that serve as AhR ligands, influencing immune homeostasis and inflammation. In IPF, aberrant tryptophan metabolism may disrupt AhR-mediated immunoregulation, contributing to persistent inflammation and fibrosis. Furthermore, imbalances in secondary bile acids, which are microbial metabolites derived from primary bile acids, have been implicated in promoting inflammation and fibrotic responses. Secondary bile acids can modulate immune cell function and inflammatory signaling pathways, and their dysregulation due to gut microbiota alterations may exacerbate lung tissue injury and fibrosis in IPF. Collectively, these microbial metabolite disturbances - reduced SCFAs, altered tryptophan metabolism impacting AhR signaling, and secondary bile acid imbalance - form a mechanistic link between gut dysbiosis and the promotion of pulmonary fibrosis in IPF [3,6,10-12].

Immune System Regulatory Mechanisms

Gut microbiota dysbiosis profoundly influences systemic and local immune responses, which are critical in the pathogenesis of IPF. One key mechanism involves the disruption of regulatory Treg function. Tregs are essential for maintaining immune tolerance and suppressing excessive inflammation. Dysbiosis can lead to impaired Treg differentiation and function, resulting in unchecked immune activation and chronic inflammation that favor fibrogenesis. Concurrently, the balance between pro-inflammatory Th17 cells and anti-inflammatory Tregs is disturbed, with an increased Th17/Treg ratio promoting the release of pro-fibrotic cytokines such as IL-17 and TGF-β. This imbalance contributes to the recruitment and activation of fibroblasts and myofibroblasts, driving extracellular matrix deposition and lung fibrosis. Moreover, gut microbiota components can activate innate immune responses through Toll-like receptors (TLRs), especially TLR2 and TLR4, expressed on epithelial and immune cells. Activation of TLRs by microbial-associated molecular patterns leads to NF-κB signaling and production of inflammatory cytokines, perpetuating tissue injury and fibrosis. In IPF, aberrant TLR signaling exacerbates inflammation and fibrotic remodeling. Therapeutic modulation of these immune pathways, including restoring Treg function, rebalancing Th17/Treg ratios, and inhibiting TLR-mediated inflammation, represents a promising strategy to mitigate IPF progression [3,11,12].

Gut-Lung Axis Signaling Pathways

The gut-lung axis constitutes a bidirectional communication network wherein gut microbiota-derived metabolites and immune cells influence the pulmonary microenvironment, and vice versa. Microbial metabolites such as SCFAs and secondary bile acids enter systemic circulation and reach the lungs, modulating local immune responses and tissue homeostasis. These metabolites can regulate alveolar macrophage function, epithelial barrier integrity, and inflammatory signaling, thereby impacting IPF pathogenesis. Additionally, immune cells primed in the gut-associated lymphoid tissue, including Tregs and Th17 cells, can migrate to the lung and participate in local immune regulation or fibrosis. The trafficking of these immune cells is influenced by chemokines and adhesion molecules, linking gut immune status to pulmonary inflammation. Another critical component of gut-lung communication is the vagus nerve, which provides a neuroimmune pathway facilitating rapid signaling between gut and lung tissues. Vagal nerve stimulation modulates inflammatory responses via the cholinergic anti-inflammatory pathway, influencing cytokine production and immune cell activation in the lungs. Dysregulation of this neural pathway may contribute to exaggerated inflammation and fibrosis in IPF. Therefore, the gut-lung axis integrates microbial, immune, and neural signals that collectively regulate lung fibrosis development, offering multiple targets for therapeutic intervention [3,6,10].

Potential therapeutic strategies and research prospects

Probiotics and Prebiotics Intervention

The clinical application prospects of specific probiotic strains such as Bifidobacteria and Lactobacilli in IPF are promising due to their well-documented roles in modulating gut microbiota and immune responses. These probiotics have demonstrated the ability to restore microbial balance and produce beneficial metabolites like SCFAs, which exert anti-inflammatory and immunomodulatory effects relevant to fibrotic lung diseases [3,13]. For instance, Lactiplantibacillus sp. LP03 was shown to alleviate pulmonary fibrosis in a bleomycin-induced mouse model by remodeling gut microbiota and elevating systemic palmitoylethanolamide levels, which suppressed TGF-β1/Smad2/3-mediated EMT, a key fibrogenic pathway [11]. This indicates that targeted probiotic therapy can influence lung pathology via the gut-lung axis. The supplementation of dietary fibers, which serve as prebiotics, holds potential in modulating gut microbial metabolism by promoting the growth and activity of beneficial bacteria. Prebiotics such as inulin, pectin, and xylooligosaccharides have been shown to alter immunomodulatory properties of probiotics, influencing cytokine profiles and regulatory T cell populations, which could be harnessed to attenuate inflammation and fibrosis [14]. Moreover, the incorporation of prebiotics in functional foods can enhance the viability and efficacy of probiotics, thereby optimizing therapeutic outcomes [15,16]. The development of personalized probiotic combinations tailored to individual microbiota compositions and disease phenotypes represents a future direction in IPF management. Advances in microbiome-based precision nutrition emphasize the importance of strain-specific effects, dosage, and treatment duration to maximize benefits and minimize adverse effects [17,18]. Collectively, these findings underscore the translational potential of probiotics and prebiotics as adjunctive therapies in IPF, warranting further clinical trials to establish standardized protocols and elucidate mechanistic pathways.

FMT Application

FMT has emerged as a novel therapeutic approach aimed at restoring gut microbial homeostasis, with preliminary evidence supporting its safety and efficacy in respiratory diseases including IPF. Although direct clinical trials in IPF remain limited, FMT has demonstrated beneficial effects in modulating systemic inflammation and immune responses in related pulmonary conditions, suggesting potential applicability [1,19]. The safety profile of FMT is generally favorable; however, rigorous donor screening is imperative to prevent pathogen transmission and ensure microbial compatibility. Current challenges include establishing standardized donor selection criteria, optimizing transplantation protocols (such as dosing, frequency, and delivery routes), and monitoring long-term outcomes [19]. The heterogeneity of gut microbiota among individuals necessitates personalized approaches to maximize therapeutic efficacy. Moreover, combining FMT with established antifibrotic agents or other pharmacotherapies may yield synergistic effects by concurrently targeting microbial dysbiosis and fibrotic pathways. For example, integrating FMT with probiotics or prebiotics could enhance colonization resistance and metabolite production, thereby potentiating anti-fibrotic mechanisms [3,11]. Future research should focus on well-designed randomized controlled trials to evaluate the clinical benefits of FMT in IPF, elucidate mechanistic interactions within the gut-lung axis, and develop guidelines for safe and effective implementation.

Targeting Microbial Metabolism for Therapy

Therapeutic strategies targeting microbial metabolism represent a cutting-edge frontier in managing IPF by modulating key metabolites implicated in fibrosis. Supplementation with SCFAs, especially butyrate, has shown promise in experimental models by exerting anti-inflammatory effects, enhancing epithelial barrier integrity, and regulating immune cell function [3,20]. SCFAs can inhibit profibrotic signaling pathways and promote regulatory T cell differentiation, thereby attenuating fibrogenesis. Additionally, bile acid receptor modulators are gaining attention due to bile acids’ role as signaling molecules influencing immune responses and epithelial homeostasis. Altered bile acid metabolism and receptor signaling have been linked to immune dysregulation and fibrosis progression, suggesting that pharmacological agents targeting bile acid receptors (such as FXR and TGR5) could modulate these pathways to therapeutic advantage [3,21]. Furthermore, novel interventions aimed at the tryptophan metabolic pathway, which produces AhR ligands involved in fibrogenesis, offer new avenues for therapy. Dysregulated tryptophan metabolism contributes to immune imbalance and tissue remodeling in IPF, and targeting this pathway may mitigate fibrosis by modulating AhR signaling [22]. Collectively, these metabolite-focused strategies underscore the importance of integrating microbial metabolic modulation with conventional therapies. Future research should prioritize mechanistic studies and clinical trials to validate these approaches, optimize dosing, and assess long-term safety, ultimately advancing personalized medicine in IPF treatment.

## Conclusions

In conclusion, this review consolidates evidence that gut microbiota dysbiosis plays a significant role in the pathogenesis of IPF through key mechanisms such as altered microbial metabolite production, immune dysregulation characterized by Th17/Treg imbalance, and disrupted gut-lung axis communication. These findings affirm that intestinal microbial imbalance is an active contributor to pulmonary fibrotic processes, repositioning IPF within a systemic framework that extends beyond the lung.

Therapeutically, interventions aimed at restoring microbial homeostasis, including probiotics, prebiotics, and FMT, offer promising adjunctive potential. Moving forward, rigorously designed clinical trials and personalized approaches informed by individual microbiome signatures will be essential to translate these insights into effective treatment strategies for IPF patients.
